# Photocatalytic Degradation of Mecoprop and Clopyralid in Aqueous Suspensions of Nanostructured N-doped TiO_2_

**DOI:** 10.3390/molecules15052994

**Published:** 2010-04-27

**Authors:** Daniela Šojić, Vesna Despotović, Biljana Abramović, Nadia Todorova, Tatiana Giannakopoulou, Christos Trapalis

**Affiliations:** 1 Department of Chemistry, Faculty of Science, University of Novi Sad, Trg D. Obradovića 3, 21000 Novi Sad, Serbia; E-Mails: daniela.sojic@dh.uns.ac.rs (D.Š.); vesna.despotovic@dh.uns.ac.rs (V.D.); 2 Laboratory of Nanocomposite and Nanofunctional Materials, IMS, NCSR Demokritos, Athens 153 10, Greece; E-Mails: todorova@ims.demokritos.gr (N.T.); tgia@ims.demokritos.gr (T.G.); trapalis@ims.demokritos.gr (C.T.)

**Keywords:** N-doped TiO_2_, mecoprop, clopyralid, photocatalysis, visible, UV light

## Abstract

The work describes a study of the oxidation power of N-doped and undoped anatase TiO_2_, as well as TiO_2_ Degussa P25 suspensions for photocatalytic degradation of the herbicides *RS*-2-(4-chloro-*o*-tolyloxy)propionic acid (mecoprop) and 3,6-dichloro-pyridine-2-carboxylic acid (clopyralid) using visible and UV light. Undoped nanostructured TiO_2_ powder in the form of anatase was prepared by a sol-gel route. The synthesized TiO_2_, as well as TiO_2_ Degussa P25 powder, were modified with urea to introduce nitrogen into the structure. N-doped TiO_2_ appeared to be somewhat more efficient than the starting TiO_2_ (anatase) powder when visible light was used for mecoprop degradation. N-doped TiO_2_ Degussa P25 was also slightly more efficient than TiO_2_ Degussa P25. However, under the same experimental conditions, no degradation of clopyralid was observed in the presence of any of the mentioned catalysts. When the kinetics of mecoprop degradation was studied using UV light, more efficient were the undoped powders, while in the case of clopyralid, N-doped TiO_2_ Degussa P25 powder was most efficient, which is probably a consequence of the difference in the molecular structure of the two herbicides.

## 1. Introduction

The photocatalytic degradation of pollutants in water and air has attracted much interest in the last several decades, as can be seen from the recent reviews [1,2,3]. Among the semiconductor photocatalysts (oxides, sulfides, *etc.*), TiO_2_ has been most extensively investigated due to its high photocatalytic activity, chemical and biological stability, insolubility in water, acidic and basic media, non-toxicity and availability. Besides, TiO_2_ photocatalysis is now being used in practical applications such as self-cleaning, sterilization, deodorizing and air-cleaning [4]. As known, photocatalysis by semiconductors is a result of the interaction of electrons and holes, generated in a solid by light absorption, with the surrounding medium. The electron-hole pairs formed in the particle can recombine or participate in reductive and oxidative reactions that can lead to the decomposition of contaminants. In an aqueous solution, the holes at the TiO_2_ surface are scavenged by surface hydroxyl groups and water molecules to generate **^·^**OH radicals. The resulting **^·^**OH radical, being a very strong oxidizing agent (standard redox potential +2.8 V) [5], can oxidize all organic compounds to the mineral final products, *i.e.* CO_2_, H_2_O and corresponding inorganic ions, if the compound contains heteroatoms.

However, an important drawback of TiO_2_ as catalyst is that its band gap is rather large, 3.0–3.2 eV, and thus only a small fraction of the solar spectrum (λ < 380 nm, corresponding to the UV region) is absorbed. The optimization of TiO_2_ structure and properties has attracted much attention, aiming to extend the absorption from the UV to visible light region and enhance the photocatalytic efficiency. Because of that, a great deal of research has been focused on doping TiO_2_ with various metals and non-metals [6]. Doping with metals has shown both positive and negative effects. Indeed, several authors have reported that although metal ion doping decreases the photo-threshold energy of TiO_2_, the metal ions may also serve as recombination centers for electrons and holes, thus reducing the overall activity of the photocatalyst [7,8,9]. However, some anionic species such as nitrogen, carbon and sulphur were identified to potentially form new impurity levels close to the valence band whilst maintaining larger band gap for maximum efficiency. Many previous works indicated that doping TiO_2_ with nitrogen is one of the most effective approaches in improving properties and photocatalytic activity of TiO_2_ in the visible light region [10,11]. Namely, N-doped TiO_2_ is reported to exhibit enhanced photoactivity under visible light irradiation due to red shift in the absorption edge.

**Figure 1:**
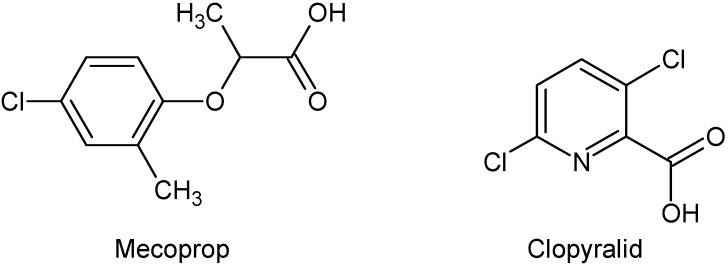
Structures of the pesticides.

The aim of this work was to investigate the efficiency of N-doped and undoped anatase TiO_2_ as well as TiO_2_ Degussa P25, as a photocatalyst in the removal of herbicides *RS*-2-(4-chloro-*o*-tolyloxy)propionic acid (mecoprop, C_10_H_11_ClO_3_) and 3,6-dichloropyridine-2-carboxylic acid (clopyralid, C_6_H_3_Cl_2_NO_2_) (Figure 1) using different UV/Vis spectral regions for irradiation. These pesticides were chosen because of their wide usage for the selective control of many annual and some perennial weeds, as well as because they are the most frequently found pesticides in drinking and stormwater [12,13]. After the characterization of the synthesized catalysts, their photocatalytic efficiency was investigated using artificial visible and UV light sources. We also aimed to investigate if differences in molecular structure influence the photocatalytic activity of the catalyst.

## 2. Results and Discussion

### 2.1. Characteristics of catalysts

**Figure 2:**
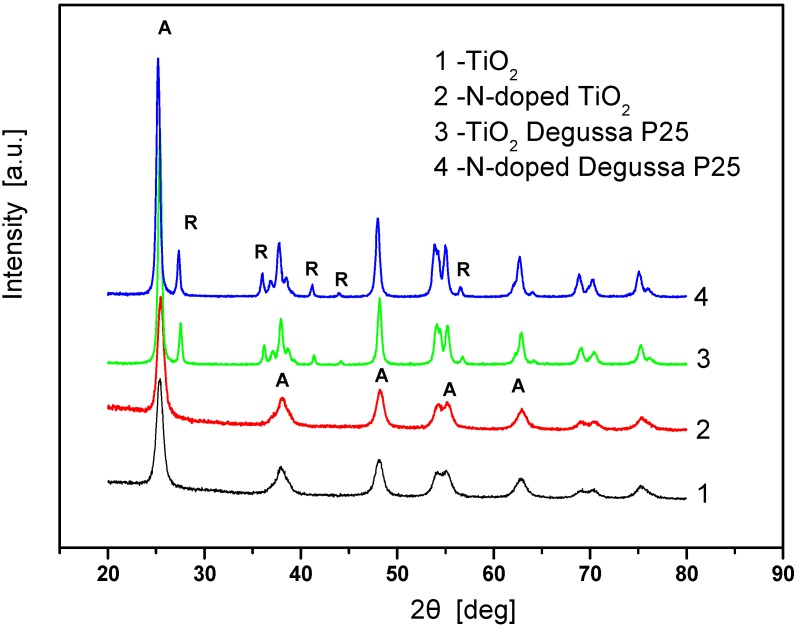
XRD patterns of undoped and urea-modified TiO_2_ powders.

The X-ray diffraction (XRD) patterns of the powders are presented in Figure 2, where A stands for anatase and R for rutile phases. It can be observed that the synthesized TiO_2_ powder consists of pure anatase (curve 1). The pattern of TiO_2_ Degussa P25 powder (curve 3) exhibited both anatase and rutile crystalline polymorphs in a wt % ratio A: R = 88: 12 (Table 1), which is in accordance with recently reported values in the literature [14,15]. After the doping procedure, the crystalline structure of each powder was preserved (curves 2 and 4) due to the temperature of thermal treatment which was only 50 °C higher than the calcination temperature of the undoped powder and below the temperature of anatase-to-rutile phase transition. In addition, the intensities and widths of the characteristic peaks for the undoped powders are similar to those of the respective N-doped samples. Only a small increase in the crystallite size, especially for the synthesized TiO_2_ was found, while the phase composition of the mixed-phase samples remained unchanged.

**Table 1:** Phase composition and average diameter of the crystallites in the studied samples.

Parameter	Nominal name			
	TiO_2_	N-doped TiO_2_	TiO_2_ Degussa P25	N-doped TiO_2_ Degussa P25
Phase composition [wt %]	A: 100	A: 100	A: 87.6	A: 87.3
			R: 12.4	R: 12.7
Average diameter [nm]	A: 9.3	A: 12.3	A: 19.9	A: 18.9
			R: 27.2	R: 28.0

**Figure 3:**
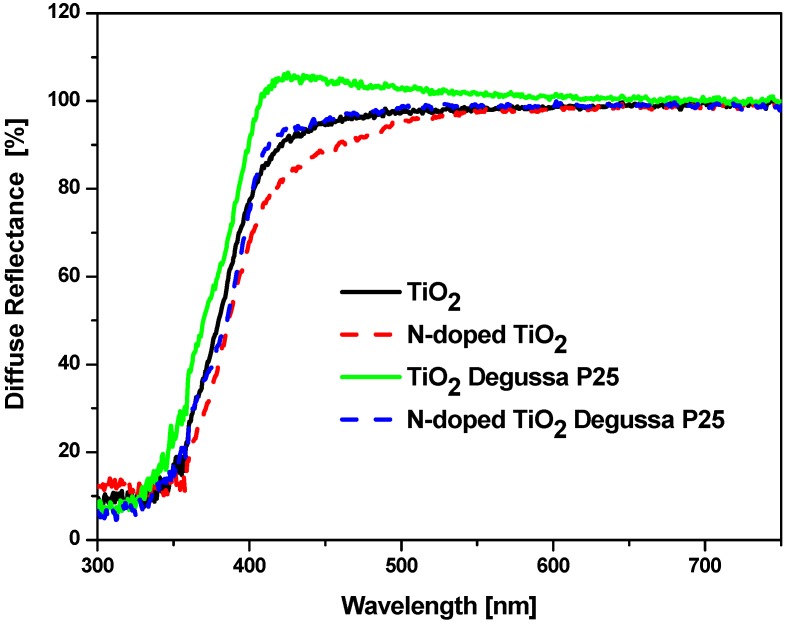
UV-Vis diffuse reflectance spectra of undoped and urea-modified TiO_2_ powders.

**Figure 4:**
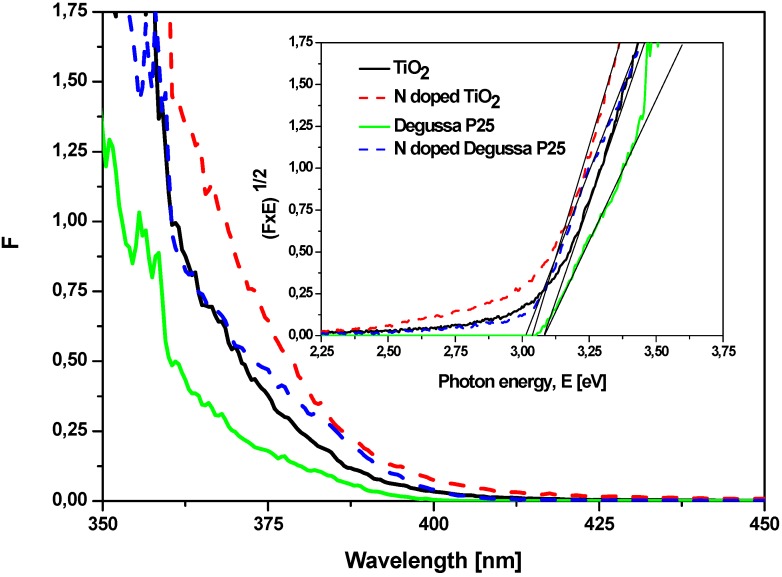
Kubelka-Munk absorption curves F for the powders studied. The band gaps appear in the inset as a result of the extrapolation procedure.

UV-Vis diffuse reflectance spectra R of the studied powders are presented in Figure 3. The Kubelka-Munk relation F = (1 − R)^2^/2R was used to convert the diffuse reflectance spectra R into the equivalent absorption spectra F (Figure 4). It can be observed that the N-doped TiO_2_ and N-doped TiO_2_ Degussa P25 powders exhibit increased absorption in the visible region in comparison to their undoped precursors TiO_2_ and Degussa P25, respectively. This is evidenced by the shift of the absorption curves to larger wavelengths and by the enhancement of the Urbach tails. The energy band gaps Eg given in the inset were obtained by extrapolation of the linear part of the absorption function (FxE)^1/2^. The band gap values are: TiO_2_ — 3.08 eV, N-doped TiO_2_ — 3.03 eV, TiO_2_ Degussa P25 — 3.08 eV and N-doped TiO_2_ Degussa P25 — 3.01 eV. Thus, the increased amount of visible light absorbed energy can be related to two phenomena: introduction of surface structural defects and lattice alteration during the modification procedure. In fact, the width of the band gap was only slightly affected by the thermal treatment in the presence of urea as the Eg values decreased only by 0.05 eV and 0.07 eV for the N-doped samples (inset of Figure 4). These findings suggest that the treatment procedure leads mainly to surface modification of the catalyst and not to incorporation of the nitrogen into its structure. Hence, the photocatalytic behavior of the catalysts is more likely to be connected to the introduction of electronic states in the band gap rather than to the band gap narrowing [16].

### 2.2. Photocatalytic activity

**Figure 5:**
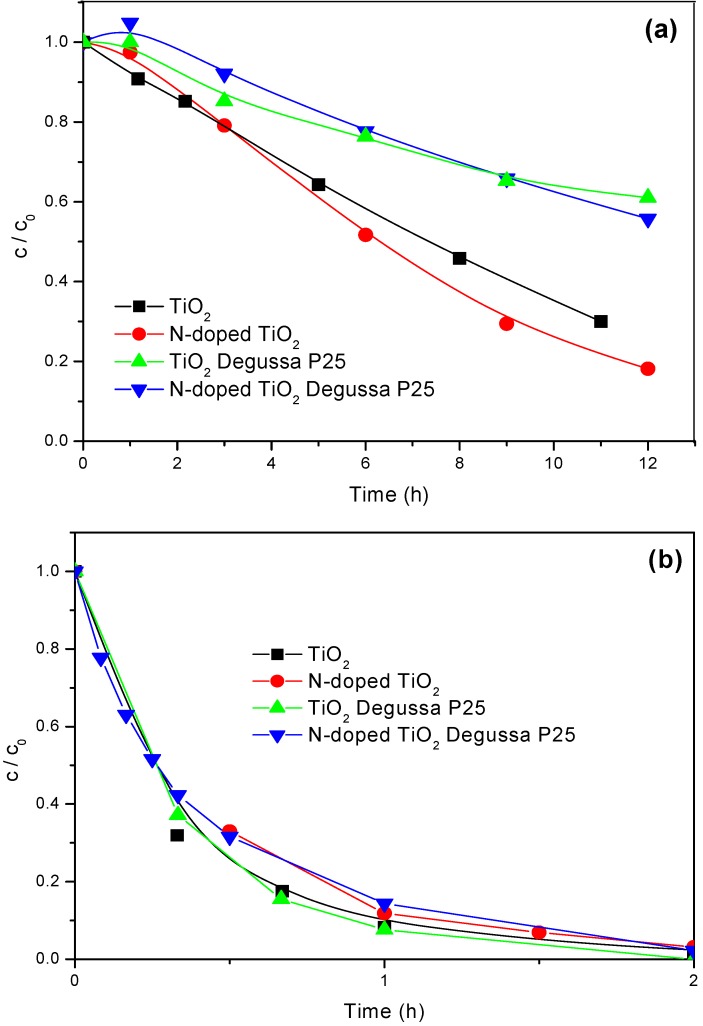
Disappearance of mecoprop (2.7 mmol/dm^3^) under visible (a) and UV (b) light irradiation (1.0 mg/cm^3^ of catalyst).

To investigate the photocatalytic activity of N-doped TiO_2_ and N-doped TiO_2_ Degussa P25, the degradation of mecoprop (Figure 5a) was carried out using artificial visible light. As can be seen, the N-doped TiO_2_ appeared to be somewhat more efficient than the starting TiO_2_ powder. Besides, N-doped TiO_2_ Degussa P25 is also slightly more efficient than TiO_2_ Degussa P25, which was more pronounced after a prolonged time of irradiation. Such behavior was expectable since, as already mentioned, the absorption edge of the doped catalysts is shifted to the visible range and higher absorption due to the alteration in the Urbach tails is observed (Figure 4). Additionally, in spite of the fact that N-doped TiO_2_ showed slightly increased value Eg in comparison with N-doped TiO_2_ Degussa P25 (in the frames of the evaluation error), the higher photocatalytic activity of the former one in the visible range can be also assigned to its higher absorption in this region which is revealed by the position of the Urbach tails (Figure 4). 

It is interesting to note that in the first period of photocatalytic degradation on the N-doped TiO_2_ Degussa P25 the concentration of mecoprop is higher than their initial equilibrium concentration, which may be ascribed to desorption of herbicide induced by irradiation [17].

Under visible light irradiation, the undoped TiO_2_ and TiO_2_ Degussa P25 exhibited high photocatalytic activity (Figure 5a) too, which is a consequence of the formation of a charge-transfer complex between the mecoprop and catalyst [18,19]. Namely, TiO_2_ treating with mecoprop caused a red shift and a slight absorbance in the visible region (400–500 nm) was observed in comparison with the spectrum of TiO_2_ powder. This indicates the formation of a charge-transfer complex between TiO_2_ and mecoprop. Taking into account the FTIR spectra, it was postulated that the charge-transfer complex between TiO_2_ and mecoprop is formed through carboxylate formation [18]. 

**Figure 6:**
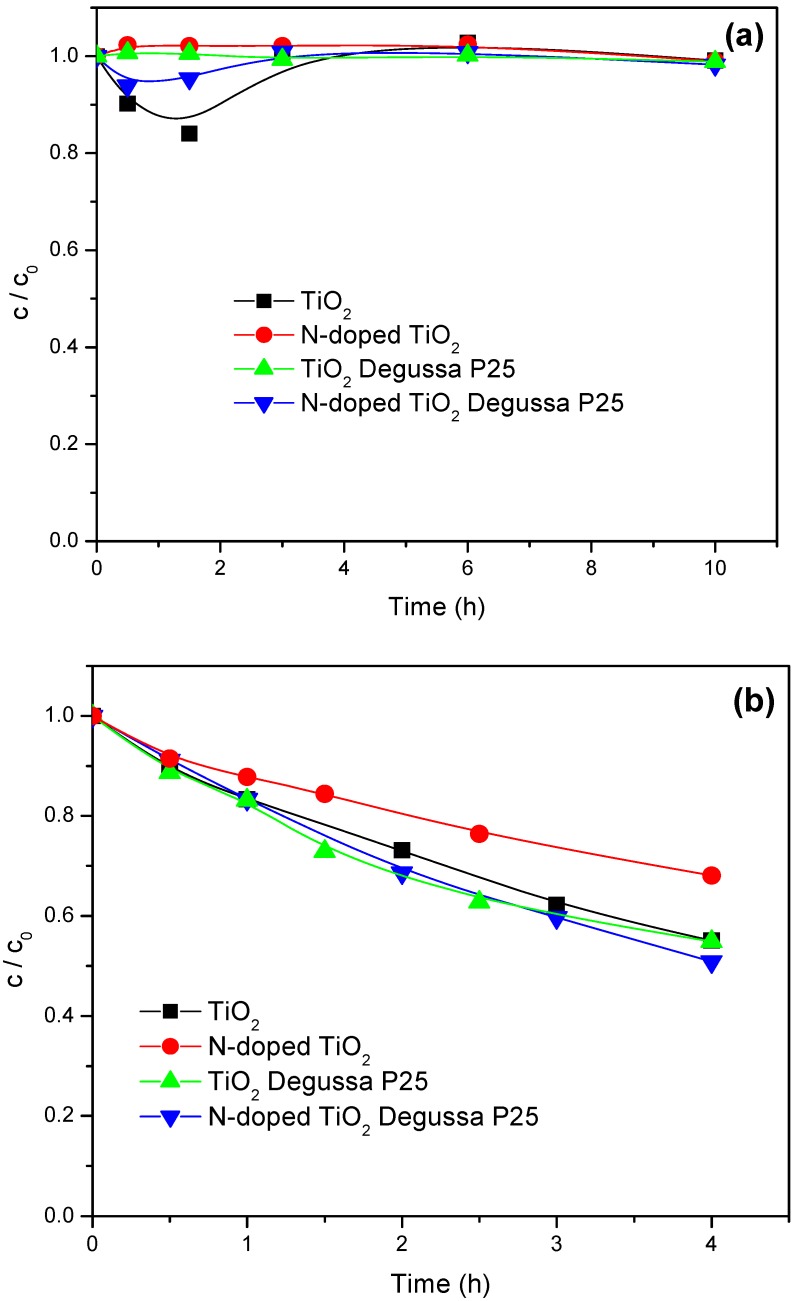
Disappearance of clopyralid (1.0 mmol/dm^3^) under visible (a) and UV (b) light irradiation (1.0 mg/cm^3^ of catalyst).

To study how the molecular structure of the pesticide substrate influences its degradation rate, we compared the degradation processes for mecoprop, containing an aromatic ring (Figure 5) and clopyralid (Figure 6), characterized by the presence of pyridine ring. 

It appeared that the situation in the case of clopyralid was quite different (Figure 6) compared to mecoprop in the presence of both types of irradiation. Namely, under the visible light, clopyralid is practically not degraded in the presence of any of the mentioned catalysts (Figure 6a), whereas under UV irradiation (Figure 6b) some degradation takes place, but at a rate which is by about three times lower than in the case of mecoprop. The photocatalytic degradation of mecoprop and clopyralid seems to be related to the difference in p*K*_a_ values (for mecoprop p*K*_a_ = 3.11–3.78 [20] and for clopyralid p*K*_1_ = 1.4 and p*K*_2_ = 4.4 [21]) [22] and to the difference in molecular structure of these compounds. The larger rate of clopyralid photodegradation under UV than under visible irradiation is in agreement with results of other authors [22], who explain it by the difference in the mechanism of the process.

The photocatalytic activities of N-doped and undoped photocatalysts were also compared using UV irradiation (Figure 5b and Figure 6b). The photocatalytic degradation of mecoprop (Figure 5b) appeared to be somewhat more efficient in the presence of undoped powders (TiO_2_ and TiO_2_ Degussa P25). However, when studying the kinetics of clopyralid degradation (Figure 6b), N-doped TiO_2_ Degussa P25 was slightly more efficient, which is also a consequence of the difference in molecular structures of the herbicides.

**Figure 7:**
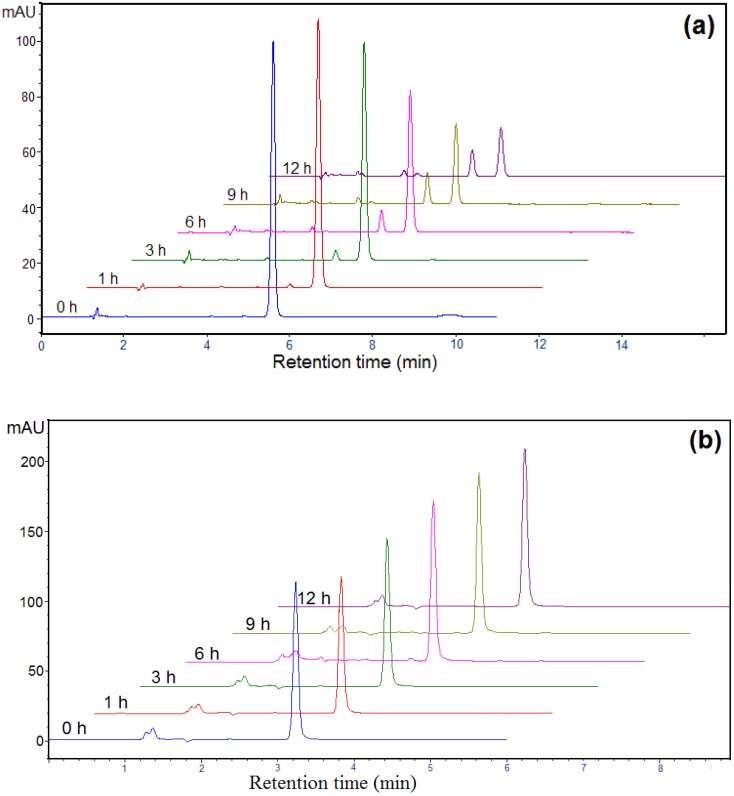
HPLC profiles during the mecoprop (2.7 mmol/dm^3^), λ = 228 nm **(a)** and clopyralid (1.0 mmol/dm^3^), λ = 225 nm **(b)** photocatalytic degradation in the presence of N-doped TiO_2_ (1.0 mg/cm^3^) using visible light.

On Figure 7, two dimensioned chromatograms obtained from photocatalytic degradation of mecoprop (Figure 7a) and clopyralid (Figure 7b) under visible light irradiation are presented. As can be seen from Figure 7a, the peak of the starting compound appears at the retention time of about 5.5 minutes and shows a decrease with irradiation time. At the same time, intermediates at 3.2, 3.5 and 4.9 minutes are formed, and these peaks increase with irradiation time. Figure 7b illustrates the degradation of clopyralid. There is only signal for the starting compound at the retention time of 3.2 minutes, which decomposes only to a very small extent, while no intermediates are registered during the process of irradiation. This was expected bearing in mind the corresponding kinetic curves (Figure 6a). 

**Figure 8:**
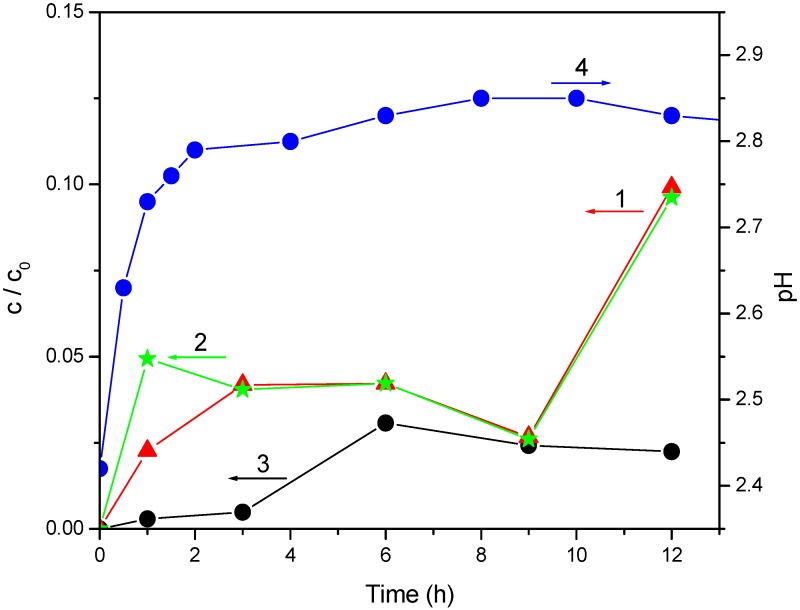
Photocatalytic degradation of mecoprop (2.7 mmol/dm^3^) in the presence of N-doped TiO_2_ (1 mg/cm^3^): (1) chloride ion formation; (2) acetate ion formation; (3) formiate ion formation and (4) pH.

In view of the fact that N-doped TiO_2_ appeared to be the most efficient as catalyst under visible light irradiation in the case of mecoprop photodegradation, ionic chromatography was employed to monitor the ionic degradation products. Since mecoprop contains covalently bounded chlorine, which is converted to chloride during photocatalytic degradation, the kinetics of chloride generation were monitored (Figure 8, curve 1). 

**Figure 9:**
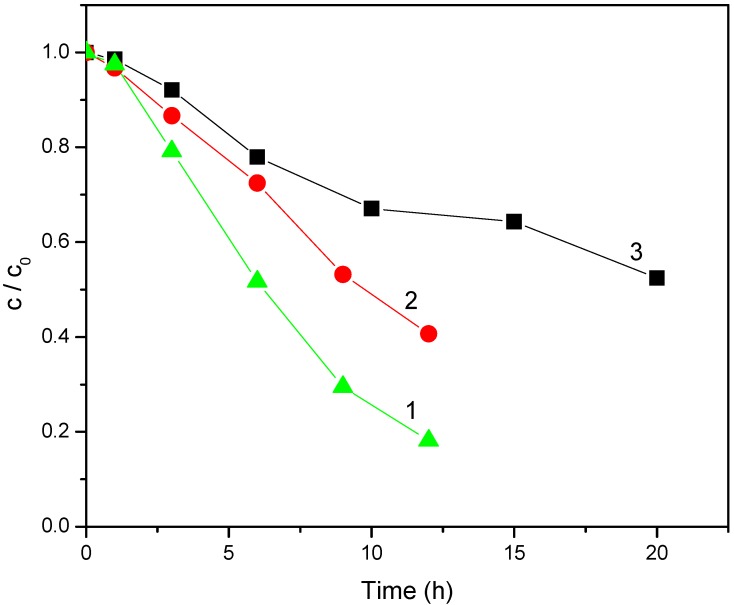
Photocatalytic degradation of mecoprop (2.7 mmol/dm^3^) in the presence of N-doped TiO_2_ (1 mg/cm^3^): (1) disappearance of mecoprop (HPLC, λ = 228 nm); (2) disappearance of aromatic ring (spectrophotometry, λ = 228 nm) and (3) total organic carbon (TOC).

After 12 h a small quantity of chloride was registered. The reaction of chloride release is slower compared to that of mecoprop (Figure 9, curve 1), as well as to aromatic ring (Figure 9, curve 2) degradation. This suggests formation of aliphatic intermediates containing Cl, which indicates that the degradation mechanism of mentioned compound under visible light irradiation is different from that under UV irradiation [18,23]. Also, the slope of the spectrophotometrically obtained kinetic curve was smaller compared to that obtained by HPLC (Figure 9, curve 1), which is understandable if we bear in mind that spectrophotometry monitored the kinetics of degradation of the aromatic ring (mecoprop and its degradation products with aromatic ring), whereas HPLC (at the retention time of 5.5 minute) measured only the change of mecoprop concentration. Besides, the rate of mecoprop degradation in the presence of the N-doped TiO_2_ (Figure 9, curve 2) whose synthesis is described in the present work, was compared to the rate measured in the presence of the catalyst obtained by mixing ammonia with titanium tetraisopropoxide [19]. It was found that the 12-hour irradiation resulted in the degradation of 60% and 35%, respectively, *i.e.*, the N-doped TiO_2_ synthesized in this work was significantly more efficient. Monitoring of the kinetics of photocatalytic degradation via the change in the pH has mainly been investigated for simple molecules, where practically no intermediates are formed, and therefore the formation of hydronium ions directly corresponds to the kinetics of degradation of the initial compound [24]. This is generally not the case with more complex molecules, where the change in pH cannot be used for kinetic analysis, but even so, its monitoring during a photocatalytic process gives a valuable information about the changes in the investigated system. Thus, the loss of carboxylic group in the initial phase of photodegradation led to the formation of less acidic intermediates, which caused a smaller increase in the pH (Figure 8, curve 4). Hence, it can be concluded that no HCl is released in this period. Under these experimental conditions acetate and formiate are formed (Figure 8, curves 2 and 3, respectively), yielding CO_2_ and H_2_O. Hence, the final degradation products are CO_2_, H_2_O and HCl. These data are in agreement with those in the literature [23]. TOC method showed a mineralization degree of mecoprop of 50% for 20 hours of irradiation (Figure 9, curve 3).

### 2.3. Effect of substrate structure on photocatalytic activity

**Figure 10:**
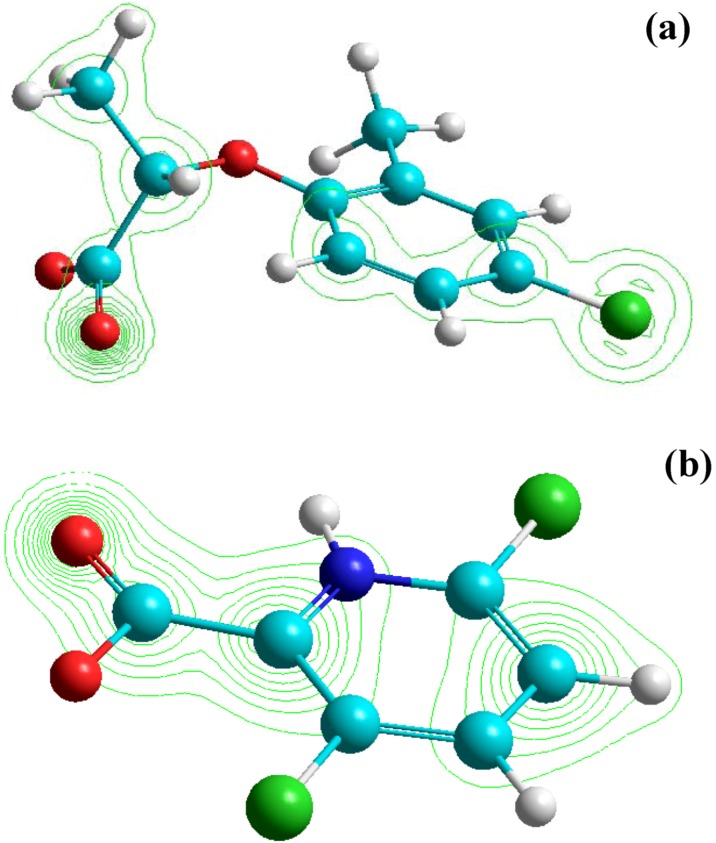
TCD maps for the herbicide molecules: mecoprop (a) and clopyralid (b). TCD contours are shown in green lines, C atoms - cyan, H atoms - white, O atoms - red, Cl atoms - green and N atom - blue.

The differences in the degradation rate of substrates is directly related to the electron-donor or electron-withdrawing character of the different substituents in the herbicide aromatic/pyridine ring, which can activate or deactivate the ring with respect to the electrophilic attack of the **^·^**OH radical. This may be explained in terms of the effect of Cl and CH_3_ group as substituents. Namely, the higher reactivity of mecoprop compared to clopyralid is probably due to the presence of the benzene-ring activating CH_3_ group (Figure 1). The mecoprop molecule contains also Cl atom, playing a deactivating role. However, the clopyralid molecule has two Cl atoms bound to the pyridine ring, causing its lower reactivity compared to that of mecoprop. These results are in agreement with those reported in the literature [25]. Also, it is known that pyridine, because of the presence of N atom in its ring, is less reactive than benzene, leading to lower reactivity of clopyralid compared to mecoprop. Besides, the presence of the electronegative COO^−^ group, clearly shown in the Hyperchem-derived total charge density (TCD) (Figure 10), indicates that the bond between the substrate and the catalyst is realized via the COO^−^ group in the case of both herbicides, bearing in mind that the pH of the suspension is about 3, which renders the catalyst surface positively charged. Figure 10 shows that the charge density on the COO^−^ group of mecoprop is higher compared to that of clopyralid, this property being directly proportional to the photoreactivity of the herbicide molecule.

The herbicides adsorption on TiO_2_ Degussa P25 was also assessed to determine how herbicide structure influences the adsorption on the catalyst. It appeared that the adsorption after 15-minute sonification of clopyralid suspension was very low (about 3%), the adsorption of mecoprop being somewhat more pronounced (about 6%). This is directly proportional to the photocatalytic degradation rate of the mentioned herbicides and may be still another explanation for the slower reaction of clopyralid compared to mecoprop.

## 3. Experimental Section

### 3.1. Chemicals and solutions

The commercial herbicide mecoprop (98% purity), obtained from the Chemical Factory “Župa” Kruševac, Serbia, was purified by conventional recrystallization from water−ethanol (1:1, v/v) solution. The purity of this purified mecoprop was confirmed by ^1^H-NMR spectrometry (Bruker AC-250). The herbicide clopyralid (99.4%), Pestanal quality, purchased from Riedel-de Haën, was used without further purification, as well as other chemicals used. For all experiments, mecoprop solutions of the initial concentration of 2.7 mmol/dm^3^ were prepared in doubly distilled water, whereas the initial concentration of clopyralid was 1.0 mmol/dm^3^. All experiments were carried out using a 1 mg/cm^3^ suspension of catalyst. Acetonitrile (ACN, 99.8%) was a product of J.T. Baker. The Ti and nitrogen precursors tetraethylorthotitanate (Merck) and urea (Merck), as well as the commercial TiO_2_ powder P25 (Degussa), were used as received.

### 3.2. Preparation and characterization of catalysts

TiO_2_ powder was synthesized by the sol-gel route using tetraethylorthotitanate as a Ti precursor and distilled water as a hydrolyzing agent in molar ratio 1:30. The water was added dropwise to the alkoxide and a white precipitate of Ti(OH)_4_ was immediately formed. The solution was aged for 24 hours under stirring at room temperature and pH 5. The slurry was dried at ~80 °C for 24 hours and calcinated at 400 °C for 1 h. The obtained powder, as well as commercial TiO_2_ Degussa P25 powder, was modified with urea by thermal treatment at 450 °C for 1 hour. Weight ratio TiO_2_:urea = 1:0.5 and a temperature of thermal treatment of the mixture 450 °C were chosen. A “Carbolite” muffle furnace with a heating rate of 5 °C/min was used. The resulting N-doped powders were light yellow.

The crystalline structure of the powders was investigated using XRD technique. Siemens D500 diffractometer with secondary graphite monochromator and CuKα radiation was used to obtain the XRD patterns of the samples. The measured 2*θ* range between 20 and 80° was scanned in steps of 0.03°/5 s. The phase content wt % and the size of the crystallites (nm) responsible for the Bragg reflection were calculated as described in reference [26].

UV-Vis diffuse reflectance spectra of the powders in the wavelength range 200–900 nm were obtained using a Shimadzu UV-2100 instrument.

### 3.3. Photodegradation procedure

**Figure 11:**
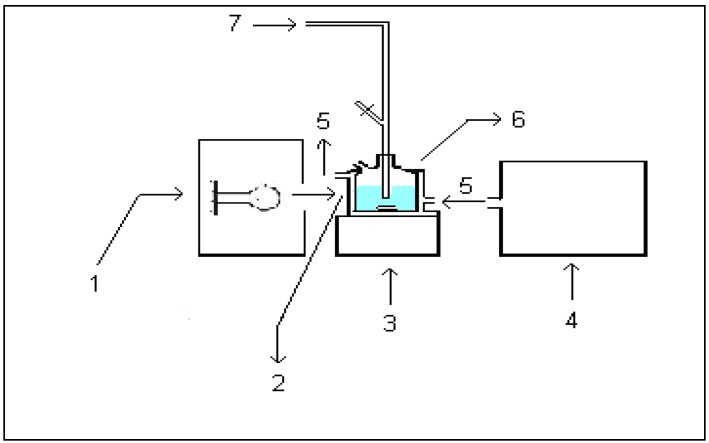
Photocatalytic reactor: (1) medium pressure mercury lamp/halogen lamp; (2) plain window of cell; (3) magnetic stirrer; (4) circulatory thermostat; (5) water; (6) photocell and (7) O_2_.

Photocatalytic reactions were carried out in a cell (sample volume 20.0 cm^3^, continuously flushed with 0.5 cm^3^/min O_2_ to serve as electron acceptor in the reaction) made of Pyrex glass with a plain window on which the light beam was focused, equipped with a magnetic stirring bar and a water circulating jacket (Figure 11). Aqueous suspensions of catalyst, containing herbicide, were sonicated for 15 min before illumination, to make the particles of photocatalyst uniform. The suspension thus obtained was thermostated at 40 ± 0.5 °C. Irradiation in the UV range was performed using a 125 W high-pressure mercury lamp (Philips, HPL-N, emission band in the UV region at 304, 314, 335 and 366 nm, with maximum emission at 366 nm), as the most commonly used light source in studying the photodegradation on TiO_2_, with an appropriate concave mirror. Besides, because of the shift of the absorption edge of N-doped TiO_2_ sample toward higher wavelengths, the irradiation in the visible range was also performed using a 50 W halogen lamp (Philips) and a 400 nm cut-off filter. The outputs for the mercury and halogen lamps were calculated to be ca. 8.8 ° 10^−9^ Einstein/(cm^3^∙min) and 1.7 ° 10^−9^ Einstein/(cm^3^∙min) (potassium ferrioxalate actinometry), respectively. During irradiation, the mixture was stirred at a constant speed. It was found that there are no losses of volatile products during the degradation. All experiments were carried out at a natural pH (~2.8 for mecoprop, and 3.2 for clopyralid).

### 3.4. Analytical procedures

For the HPLC kinetic studies of herbicide photodegradation, appropriate aliquots (0.25 cm^3^ for mecoprop and 0.5 cm^3^ for clopyralid) of the reaction mixtures were taken at the beginning of the experiment and at regular time intervals (volume variation ca. 10%), and the solution diluted to 10.00 cm^3^ with doubly distilled water. Thus obtained suspensions were filtered through Millipore (Millex-GV, 0.22 μm) membrane filter. After that, a 20-µL sample was injected and analyzed on an Agilent Technologies 1100 Series liquid chromatograph, equipped with a UV-VIS diode array detector set at 228 nm for mecoprop and 225 nm for clopyralid (maximum of absorption for the corresponding pesticide) and an Eclypse XDB-C18 (150 mm × 4.6 mm *i.d.*, particle size 5 µm, 25 °C) column. The mobile phase (flow rate 1 cm^3^/min) was a mixture of ACN and water (1:1, v/v, pH 2.68 for mecoprop and 3:7, v/v, pH 2.56 for clopyralid), the water being acidified to make 0.1% phosphoric acid. 

For the spectrophotometric monitoring, samples were prepared in the same way as for HPLC measurements and the spectra were recorded in the wavelength range from 200 to 400 nm on a Secomam Anthelie Advanced 2 spectrophotometer in 1 cm quartz cells. Kinetics of the herbicides degradation was monitored at 228 nm (mecoprop) and 225 nm (clopyralid).

For anion chromatographic determinations, aliquots of 0.25 cm^3^ of the reaction mixture were taken at regular time intervals and diluted to 10.00 cm^3^. Thus obtained suspensions were filtered through membrane filters and analyzed on a Dionex ICS 3000 ion chromatograph equipped with a Ion Pac AS18 Analytical column (250 mm × 4 mm) and a conductometric detector. The mobile phase was a solution of KOH (20−40 mmol/dm^3^), flow rate 1 cm^3^/min.

Changes in the pH during the degradation were monitored using a combined glass electrode (pH-Electrode SenTix 20, WTW) connected to the pH-meter (pH/Cond 340i, WTW).

For TOC analysis, samples were irradiated for different time intervals and analyzed on a Elementar Liqui TOC II. In all cases, correlation coefficients obtained for calibration curves were higher than 0.99.

Computer modeling procedures used in this study were performed using Hyperchem 8.0.6 (Hypercube Inc.). The compounds in the data set were entered as two-dimensional sketches into Hyperchem. Full optimization geometry and calculation of the TCD for the best conformer were performed using the semi-empirical method AM1 running on Hyperchem. Electronic properties were computed from single point calculations.

## 4. Conclusions

Thermal treatment in presence of urea was applied in order to activate TiO_2_ photocatalyst in the visible range. Enhanced absorption of the treated powders and shift towards the longer wavelengths was proved by UV-Vis spectroscopy. The results of photocatalytic degradation clearly demonstrate that the N-doped TiO_2_ powders are more efficient under visible light illumination than the starting TiO_2_ powders in the case of mecoprop, whereas in the case of clopyralid no degradation takes place in the presence of the mentioned catalysts. When UV radiation was used, photocatalytic degradation of mecoprop appeared to be more efficient in the presence of undoped catalysts (TiO_2_ and TiO_2_ Degussa P25). However, when the kinetics of degradation of clopyralid was studied, N-doped TiO_2_ Degussa P25 powder was somewhat more efficient. The results of this study clearly indicate that the N-doped TiO_2_ and undoped catalysts synthesized in this work catalyze more efficiently the disappearance of mecoprop in comparison to clopyralid, in the presence of both light sources. This can be explained by the difference in the molecular structure of the herbicides that influences the activity of the photocatalysts to a great extent.
